# NADES-Assisted Extraction of Polyphenols from Coriander Seeds: A Systematic Optimization Study

**DOI:** 10.3390/antiox12122048

**Published:** 2023-11-27

**Authors:** Federica Ianni, Samir Scandar, Luciano Mangiapelo, Francesca Blasi, Maria Carla Marcotullio, Lina Cossignani

**Affiliations:** 1 Department of Pharmaceutical Sciences, Section of Food, Biochemical, Physiological and Nutrition Sciences, University of Perugia, 06126 Perugia, Italy; luciano.mangiapelo@studenti.unipg.it (L.M.); francesca.blasi@unipg.it (F.B.); lina.cossignani@unipg.it (L.C.); 2 Department of Pharmaceutical Sciences, Section of Morphological, Biomolecular, Nutraceutical and Health Sciences (SIMBIONUS), University of Perugia, 06122 Perugia, Italy; samir.scandar@studenti.unipg.it

**Keywords:** coriander seeds, polyphenols, NADES, antioxidant activity, HPLC-DAD monitoring

## Abstract

*Coriandrum sativum* L. seeds are widely recognized for their traditional use in medicine. Among the most investigated components, the terpenoid linalool and monounsaturated petroselinic acid have attracted interest for their nutritional value. Instead, minor attention was paid to the polyphenolic fraction, resulting still being incomplete today. This study aimed to develop a systematic approach in which green natural deep eutectic solvents (NADES) were combined with conventional (maceration, MAC) or non-conventional (ultrasound-assisted extraction, UAE) techniques in a one-step methodology to recover polyphenols from coriander seeds. The NADES system choline chloride–citric acid (ChCl:CA, 1:1) was firstly evaluated, coupled with MAC or UAE, and then compared with ChCl–Urea (ChCl:Ur, 1:1) and ChCl–Glucose (ChCl:Glu, 1:1) under optimal conditions (20 min extraction time). The system ChCl:Ur UAE significantly improved the extraction of chlorogenic acid and its isomer (453.90 ± 4.77 and 537.42 ± 1.27 µg/g, respectively), while the system ChCl:Glu UAE improved the extraction of protocatechuic, caffeic and *p*-coumaric acids (131.13 ± 6.16, 269.03 ± 4.15 and 57.36 ± 0.06 µg/g, respectively). The highest levels of rutin were obtained with ChCl:CA-based NADES when the MAC technique was applied (820.31 ± 28.59 µg/g). These findings indicate that the NADES composition could be appropriately modulated to tailor extraction towards higher levels of a desirable bioactive for further applications.

## 1. Introduction

The extraction of bioactive compounds from plant products is experiencing an ever-growing trend in developing green processes in a context of sustainability and renewability. The major challenge today is represented by the reduction in volatile and flammable organic solvents, harmful to both human health and the environment, conventionally used in all of the analytical phases [1], among which the extraction is the most crucial for the isolation of the desired chemical components. In this context, numerous efforts have been made to improve extraction protocols and technology-supported approaches that would allow for a much more efficient recovery of valuable compounds than the conventional ones [2]. The ideal alternative is a low toxicity, easy to remove and environmentally friendly solvent, which could be combined with a low-energy and cost-effective, innovative technology. All these aspects assume an even more critical significance when food bioactives are considered, where the use of green solvents and more sustainable techniques allow for the production of safer products, preferentially selected by consumers.

Coriander (*Coriandrum sativum* L.) is an aromatic Mediterranean plant mainly cultivated for its seeds, which have long been used in folk medicine for the treatment of a wide range of ailments [3,4,5], and it is widely exploited as a condiment in the food industry. Furthermore, this spice finds application in the pharmaceutical field due to its numerous beneficial properties, as well as in cosmetics, proving to be a promising natural source for skin care. Essential oil, one of the seeds’ most important and studied constituents, contains linalool as the main compound and other bioactives with many potential health benefits [4]. Moreover, seeds are a source of monounsaturated fatty acids, in particular petroselinic acid (C18:1n-12) [6]. While the extraction and compositional analysis of these constituents have been extensively addressed over the years, the polyphenolic fraction of coriander seeds is still incomplete and sometimes overlooked. A recent literature analysis by Scandar et al. on *Coriandrum sativum* L. showed that over 1000 papers have been published in the last twenty years dealing with the preparation and the analysis of the essential oil, while there are only about 340 on phenols. Therefore, it would be worth investigating these compounds to gain more insights into their properties and the long history of the use of this traditional drug [5].

The well recognized properties of phenols as antiradical, antioxidant and anti-inflammatory agents and their potential therapeutic involvement in the treatment of cancer and other chronic diseases have prompted the scientific community to search for innovative techniques for the extraction of such components from several food matrices [7]. In this context, innovative extraction processes to obtain phenols from coriander seeds have been developed. Zeković et al. compared the effect of microwave-assisted extraction (MAE) with conventional maceration (MAC) on the total phenol content (TPC) and antioxidant properties [determined using a 1,1-diphenyl-2-picrylhydrazyl (DPPH) assay and a reducing power assay] of coriander extracts [8]. The same authors reported the effectiveness of supercritical fluid extraction (SFE) combined with ultrasound-assisted extraction (UAE) in extracting the non-polar and polar phenol fraction, respectively, from coriander seeds [9]. More recently, Palmieri et al. have compared different conventional and unconventional extractions of bioactives from coriander: in particular, innovative methods including ultrasound-assisted extraction (UAE) and rapid solid–liquid dynamic extraction (RSLDE) have revealed high extractive potential [10].

In recent years, natural deep eutectic solvents (NADES) are emerging as very promising solvents to enable the extraction of plant metabolites [11], organic reactions [12], biotransformations [13] and formulations [14]. NADES are a “mixture of pure compounds for which the eutectic point temperature is below that of an ideal liquid mixture” [15] and have been proven to be efficient tools for an attractive approach to antioxidant recovery. The success of NADES lies in their numerous advantages such as their low cost and easy availability, biodegradability components and low toxicity profile; moreover, they are also characterized by physicochemical properties fine-tuned for a specific purpose [16]. Their composition makes them environmentally friendly and safe to use in the food, pharmaceutical and cosmetics industries, and their use is associated with the increased stability and shelf life of extracts [17]. Nevertheless, the “greenness” of these solvents depends on their constituents [15,18]. The combination of NADES as an alternative to conventional solvents with non-conventional extraction techniques offers a great improvement in this panorama aimed at finding more efficient and sustainable processes. The literature boasts numerous examples in this sense, in which the energy released by methods based on ultrasound, heating or microwave radiation has been successfully exploited to favour the mass-transfer of target molecules in the NADES phase. Stupar et al. have optimized the recovery of β-carotene from pumpkin by exploring a series of hydrophobic NADES [19]. The system composed of caprylic acid and capric acid (3:1) was selected for the high affinity and solubility of β-carotene. The combination with a high efficiency extraction technique such as UAE intensified the process allowing a higher recovery of β-carotene (151.41 µg/mL at the optimal process conditions, compared to 96.74 µg/mL recovered with only NADES). In another study, Mansinhos et al. evaluated the effectiveness of ten different NADES, compared with conventional solvents, on the extraction of phenolic compounds from *Lavandula pedunculata* subsp. *lusitanica* (Chaytor) Franco [20]. The UAE extraction efficiency was compared to that afforded by the conventional MAC using the same NADES. Results confirmed that, in most cases, the combination with UAE improved the extraction of phenols (22.90–56.00 mg GAE/g dry weight, DW) compared to MAC (18.22–50.05 mg GAE/g DW) as well as the antioxidant properties, proving that NADES-UAE was a more effective system. A NADES-based MAE system for the extraction of phenolics from leaves of *Eugenia uniflora* L. has been recently reported. The use of choline chloride:lactic acid 1:3 (+20% water *w*/*w*) improved the extractability over hydro-ethanolic solvent, measured as the total peak area and number of peaks [21].

To the best of our knowledge, a systematic study dealing with the application of NADES-assisted (conventional or non-conventional) extraction of phenols from *Coriandrum sativum* L. is still missing in the scientific panorama. To fill this gap, this work aims to develop a new extractive approach based on the use of NADES systems formed using choline chloride as the hydrogen bond acceptor (HBA) for this plant material. Accordingly, a simple, scalable and sustainable procedure (in terms of costs, energy, time and environmental impact) is proposed. The optimization of the extraction process followed a one-variable-at-a-time (OVAT) approach was useful, at this exploratory stage, to gain peculiar information on the influence of each process variable independently. A conventional MAC extraction was firstly carried out with a traditional hydro-alcoholic solvent. Then, in the best conditions, the use of NADES as extracting solvents was evaluated and combined with MAC or the non-conventional UAE technique in a one-step methodology. In the wake of this analytical workflow, citric acid-based, urea-based and glucose-based hydrogen bond donors (HBDs) in the choline chloride-based NADES were compared under optimal conditions. Each step of the process was adequately monitored by spectrophotometry for the measurement of the total phenol content (TPC) as well as the in vitro total antioxidant capacity (TAC, determined through ABTS, DPPH and FRAP assays) and using a chromatographic (HPLC-DAD) analysis.

## 2. Materials and Methods

### 2.1. Chemicals

All the employed solvents were of analytical grade and purchased from Carlo Erba (Milan, Italy). Analytical standards were purchased from PhytoLab (Vestenbergsgreuth, Germany). Choline chloride (ThermoFisher Scientific-Alfa Aesar, Kandel, Germany), citric acid, glucose (VWR International Srl, Milan, Italy), urea (Sigma-Aldrich, Saint Louis, MO, USA) were used for the preparation of NADES. Folin–Ciocalteu reagent, 2,2′-azino-bis(3-ethylbenzothiazoline-6-sulphonic acid) diammonium salt (ABTS), (±)-6-hydroxy-2,5,7,8-tetramethylchromane-2-carboxylic acid (Trolox), 2,4,6-tris(2-pyridyl)-s-triazine (TPTZ), 2,2-diphenyl-1-picrylhydrazyl (DPPH) were from Sigma-Aldrich (Milan, Italy). Water was purified using a Milli-Q Plus 185 system from Millipore (Milford, MA, USA).

### 2.2. Plant Material

Dried seeds (fruits) of *Coriandrum sativum* L. from Bulgaria, packed under vacuum (Lot. n° 173227) by Borghini S.r.L (Arezzo, Italy), were purchased from Bavicchi Spa (Perugia, Italy). Fruits were kept at 4 °C until use, and then, they were pulverized using a Girmi-Tritatutto Mod. TR20, sieved and the <710 μm granulometric fraction collected. A voucher specimen (CS #2) was deposited at the Department of Pharmaceutical Sciences, Section of Morphological, Biomolecular, Nutraceutical and Health Sciences (SIMBIONUS), University of Perugia.

### 2.3. NADES Preparation

All solid NADES components were dried on P_2_O_5_ under vacuum for 3 h before use, and choline chloride was dried at 65 °C under vacuum for 24 h. Hydrogen bond acceptor (ChCl) and hydrogen bond donor (glucose, glycerol, urea and citric acid) in 1:1 molar amount were separately weighted, mixed in a 500 mL round-bottom flask and stirred for a few minutes. Then, water (40%, *w*/*w*) was added, and the stoppered flask was transferred into an ultrasound (US) bath Branson 2800 (130 W) (Emerson Italia, Cinisello Balsamo, Milan, Italy). The temperature was maintained constantly at 80 °C using a KitchenBoss Sous Vide G310 probe, and the sample was irradiated for 30 min. After this period, all the NADES were formed as homogeneous, viscous, transparent liquids that were stored in a desiccator equipped with dry CaCl_2_, under vacuum, until reaching constant weight. All the prepared NADES were characterized by ATR FT-IR spectra obtained using an FT-IR Shimadzu IR-8000 instrument (Shimadzu Italia, Milan, Italy) (Supplementary Materials Figures S1–S7). The spectral range was set between 400 and 4000 cm^−1^, and the spectral resolution was set at 4 cm^−1^ and 100 scans [22].

### 2.4. Extraction of Coriander Seeds by Traditional Maceration (MAC)

Pulverized coriander seeds (0.25 g) were suspended in 5 mL of aqueous MeOH (70%) (1:20 plant-to-solvent ratio) or choline chloride-based NADES and stirred at room temperature in a stoppered flask for 40 or 20 min, 2, 4 and 6 h. After the appropriate time, the extracts were filtered on a paper filter (Quantitative filter paper 474, VWR, International Srl, Milan, Italy) and then evaporated.

### 2.5. Ultrasound-Assisted Extraction (UAE) of Coriander Seeds

Pulverized coriander seeds (0.25 g) were suspended in 5 g of the chosen NADES and irradiated at 70 W, using a US apparatus (VEVOR Ultrasonic Cleaner, Model TH-10A (70 W), Jinan, China) at 25 °C for 30 min. To keep the temperature constant, a recirculating water system, in which the water was externally maintained at the desired temperature using a KitchenBoss Sous Vide G310 probe, was created using two Anself brushless aquarium pumps. After this time, the suspension was centrifuged (4236A centrifuge, ALC, Cologno Monzese, Milan, Italy) at 8000 rpm for 15 min. The supernatant was filtered using a syringe filter (0.45 μm, 30 mm, ThermoFisher Scientific-Alfa Aesar, Kandel, Germany).

### 2.6. Spectrophotometric Total Phenol Content (TPC) and In Vitro Antioxidant Activity

Spectrophotometric assays were performed according to our previous papers with slight modifications [23].

TPC was determined using the Folin–Ciocalteu method. The phenolic content of the extracts obtained in traditional conditions (hydro-methanolic mixture) was determined by measuring the absorbance at 765 nm. TPC in NADES extracts was determined following a protocol modified by Eleni Boli et al. [24]. Briefly, an aliquot of 50 µL of the extract dissolved in water/methanol (1:1, *v*/*v*) was treated with 1.5 mL of NaOH solution (4% *w*/*v*), 4.0 mL of deionized water and 25 µL of Folin–Ciocalteu reagent. Once mixed, 750 µL of aqueous sodium carbonate (20% *v*/*v*) were added and the mixture was heated in a water bath at 40 °C for 30 min. At the end of the reaction time, the absorbance was measured at 765 nm. In both protocols, the TPC value was determined by relying on a calibration curve of gallic acid (prepared as the corresponding extracts) and results were expressed as mg of gallic acid equivalents per gram of dry coriander seeds (mg GAE/g DW).

Ferric reducing power, determined via FRAP assay, was determined by incubating the extracts with the Fe^3+^-TPTZ complex for 30 min. Absorbance was measured at 593 nm. The free radical-scavenging activity was measured using DPPH and ABTS assays. The DPPH reagent was added to the extracts, and the absorbance measured at 517 nm, after 30 min incubation of the mixture. ABTS reagent was prepared, added to the extracts and the resulting mixtures were kept in the dark for 6 min before measuring the absorbance at 734 nm.

Total antioxidant capacity (TAC) was quantitatively determined using a calibration curve of Trolox, and the results, for the three assays, were expressed as mg of Trolox equivalent per gram of dry coriander seeds (mg TE/g DW). All the spectrophotometric measures were recorded with a LAMBDATM UV-Vis spectrophotometer (PerkinElmer, Inc; Waltham, MA, USA).

### 2.7. HPLC-DAD Analysis

The HPLC-DAD method was optimized and applied to evaluate the phenolic qualitative and quantitative profile of coriander seed extracts. The HPLC measurements were made on a Thermo Separation low-pressure quaternary gradient pump system coupled to a Spectra system UV 6000 LP diode array detector (DAD) (Thermo Scientific, Waltham, MA, USA), supplied with a GT-154 vacuum degasser (Shimadzu, Kyoto, Japan), and a Rheodyne7725i injector (Rheodyne Inc., Rohnert Park, CA, USA) with a 20 μL stainless steel loop. Data acquisition was completed using the Xcalibur software (version 1.1, Thermo Finnigan Company, Chromatographic Specialties Inc., Brockville, CA, USA). The column HyperSil GOLD™ C18 column (250 × 4.6 mm, 5 μm particle size, 100 Å pore size, by Thermo Fisher Scientific, Waltham, MA, USA) was conditioned for 20 min before use with the selected mobile phase.

The following best gradient program was applied: eluent A (water/formic acid, 0.1% v) and eluent B (MeOH): 0 min—100% A; 25 min—70% A; 40 min—60% A; 55 min—30% A; 70 min—100% A. The flow rate was set at 0.7 mL/min and the column temperature at 25 °C. For the quantification of each analyte identified in the extracts, calibration curves were built up using the corresponding standard solutions. The concentration was determined at the maximum wavelength of absorbance in order to have the greatest sensitivity. Therefore, a wavelength of 250 nm was used for protocatechuic acid and rutin quantification, 300 nm for *p*-coumaric acid and 330 nm for chlorogenic acid (and its isomer) and caffeic acid.

The linearity of the calibration curves is expressed by the high R^2^ values (>0.998) as reported in Table S1 (Supplementary Materials) for all the investigated species.

The established HPLC method was validated, at a basic “research” level, in terms of accuracy, precision, limit of detection (LOD) and limit of quantification (LOQ).

The three standard compounds chlorogenic acid, caffeic acid and *p*-coumaric acid were selected for method validation and two different control solutions with nominal concentrations of 3.3 and 33 μg/mL were used. The obtained results, reported in Tables S1 and S2 (Supplementary Materials), revealed the adequacy of the analytical method to be applied for quantitative purposes. High recovery % (in the range 98.07–103.31 and 99.07–101.83) and low RSD % (in the range 1.7–3.4 and 1.52–2.82) values were obtained for the evaluation of the short-(intra-day) and long-term (inter-day) accuracy and precision, respectively.

### 2.8. Statistical Analysis

All samples were prepared in triplicate. The results are expressed as mean value ± standard deviation (*n* = 3). Statistically significant differences among group means were evaluated using one-way analysis of variance (ANOVA) followed by post hoc Tukey’s for multiple comparison tests. OriginPro 9.0 (OriginLab Corporation, Northampton, MA, USA) was used as statistical software. The significance of the results was considered at *p*-value < 0.05.

## 3. Results and Discussion

### 3.1. Conventional Extraction of Phenols from Coriander Seeds

The extraction of the phenolic fraction from coriander seeds was systematically optimized using an OVAT approach. As a preliminary stage of the study, MAC as a conventional extraction method was applied with the use of the conventional organic solvent methanol and water (70:30, *v*/*v*).

Since the yield and properties of the target compounds depend on a combination of different parameters, MAC conditions need finely tuned operational requirements for each kind of extract to achieve the optimal properties. Generally, among the most influencing variables, extraction time and temperature play a key role. If on the one hand, temperature favours the extraction by enhancing phenolic compounds solubility, on the other, it can lead to their denaturation [25]. More contradictory data concern the extraction length, but in general, prolonged times could decrease the phenolic content as a result of a major exposure to environmental factors such as light and oxygen [26]. Taking these considerations into account, we focused our attention on the MAC time, by varying the length of extraction from 40 min to 6 h in order to evaluate the phenolic concentration, antioxidant activities and composition and select the best conditions for the successive optimization steps.

A controversial result emerged from the spectrophotometric assays. In fact, while 40 min allowed for obtaining the highest phenol content, an extraction time of 6 h offered the highest TAC values for the three assays (Table 1). Data reported by Palmieri et al. highlighted an improved TPC value for coriander extracts with longer extraction times [10]. However, the total phenol content measured at 40 min in this study is in line with the value found by Sriti et al., equal to 15.55 mg GAE/g DW in seeds [27]. Noteworthy, the applied mild conditions of MAC underline the gain in terms of the process time and energy consumption compared to the more drastic ones reported by the authors, where sonication for 15 min and reflux in a water bath at 90 °C for 2 h were used for phenol extraction.

The evaluation of the chromatographic results did not evidence any particular significant difference among the extracts in terms of the qualitative and quantitative profile of the identified phenols (Table 2). Rather, if the quantitative result is considered as a whole, 40 min MAC afforded the highest total content (1311.18 µg/g), followed by 2, 4 and 6 h (1213.08, 1198.10 and 1188.07 µg/g), respectively. Therefore, in the light of the obtained results, there are apparently no conditions that can justify the increased TAC value at 6 h, unless interfering species form (undetected compounds), which could take part in the reaction mechanism and have a stronger impact over the antioxidant response [28].

**Figure 1:**
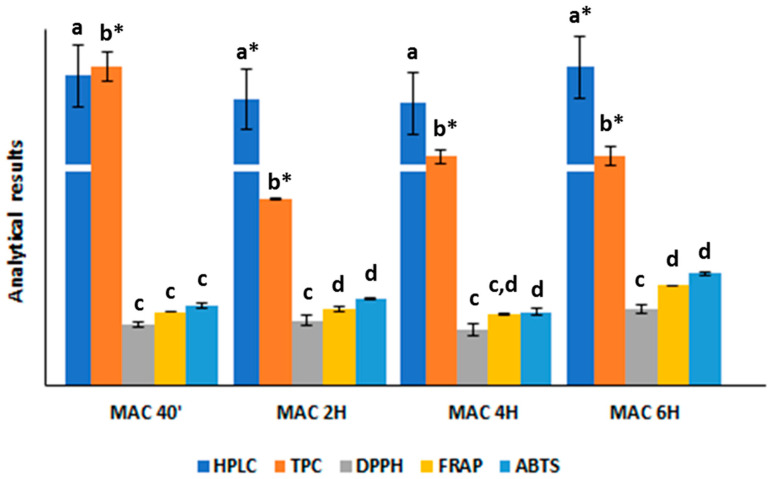
Trend of the total phenol concentration (expressed as mg GAE/g) and antioxidant activity (expressed as mg TE/g) measured using spectrophotometry, and HPLC-PC results (expressed as µg/g) relative to the coriander extracts obtained via MAC with methanol:water (70:30, *v*/*v*) as extraction solvent. Error bars in the bar graph indicate data variability for the reported measurements. Within each group corresponding to a specific extraction condition, different letters above the bars indicate a statistically significant difference at a *p*-value < 0.05 (*n* = 3). Asterisks indicate a statistically significant difference at a *p*-value <0.0001.

The total content of the phenols identified and measured using HPLC refers to the sum of the individual concentration of the compounds measured at the selected wavelength (HPLC phenol content, HPLC-PC). Due to the satisfactory correlation between TPC and HPLC-PC results (R^2^ = 0.7349) and to the higher values obtained with a MAC of 40 min, readily viewable in Figure 1, this was determined as the optimal extraction time.

### 3.2. NADES-Assisted Extraction of Phenols from Coriander Seeds by Conventional MAC and Unconventional UAE

To further improve the extraction efficiency while increasing the “greenness” of the extraction process, the ability of NADES to form hydrogen bonds through donation and acceptance of protons and electrons, allowing for increased phenol solubility, was exploited.

The amount of water is a crucial point to achieve tailored physicochemical properties of NADES such as viscosity, polarity and density. Therefore, the addition of water, commonly between 5% *w*/*w* and 50% *w/w,* can regulate the mass transfer of plant secondary metabolites and, thus, the extraction efficiency [29,30]. An excess of water, instead, has a detrimental effect on the supramolecular structure of NADES and on the intermolecular interaction with the target components. For this reason, the percentage of water in the NADES system explored in this stage of the work, based on choline chloride–citric acid (ChCl:CA, 1:1), was properly tuned, reaching the highest efficiency at 40% [31].

It has already been reported that acidic NADES exhibited higher dissolution effects on cell-wall cellulose, allowing a better extraction of metabolites [32].

The use of ChCl:CA as the NADES solvent, at different molar ratios, finds some application in the literature for the extraction of phenol compounds, i.e., from olive leaves using the conventional heating extraction procedure [33], from grape-pomace using UAE [34] and from coffee husks using MAC [35], to name a few. Some of these studies confirm that ChCl:CA provided an extraction efficiency and antioxidant activity comparable to the conventional solvents and in most cases even outperforming them.

In this frame, the system ChCl:CA, preliminarily used as solvent in place of the hydro-alcoholic mixture, coupled with MAC extraction for 40 min, did not produce any substantial variation in the TPC content of the extract (Table 3 vs. Table 1, first row). On the contrary, regarding the free radical-scavenging activity, an improvement was observed for DPPH but not for the ABTS. Analogously, the applied extraction conditions produced a reduction in terms of the reducing capacity (FRAP assay).

Taking into account the results obtained so far, namely the extraction time of 40 min and the use of ChCl:CA as the extracting solvent, the UAE technique was evaluated as a non-conventional alternative to overcome some intrinsic limitations commonly belonging to the MAC extraction. As evident from Table 3, a general improvement of the spectrophotometric assays was obtained. It should be noted that the significant contribution of polyphenol compounds to the antioxidant activity was established by performing the same assays on the NADES system alone (blank). Contrary to what was expected, given that citric acid itself is endowed with prominent antioxidant properties, no activity was measured using DPPH, FRAP and ABTS.

By exploiting one of the main advantages of the UAE based on the triggering of physico-chemical phenomena, fundamentally different than those applied in conventional extraction techniques, with an enhanced mass transfer in reduced times, a further reduction in the extraction time to 20 min was evaluated for UAE and, for comparison purposes, also applied to MAE. The results in Table 3 show a TPC value comparable to the one obtained via UAE carried out for 40 min, while a concomitant increase was not evidenced for the TAC values.

Concerning the qualitative and quantitative profile of phenols in coriander seeds, data in the literature [10,36] showed the detection of phenolic acids including gallic, *p*-hydroxy-benzoic, chlorogenic, vanillic, caffeic, syringic and ferulic acids and the flavonoids quercetin, luteolin, apigenin and rutin. The extraction conditions applied in this work allowed the HPLC identification and quantification of some of the above phenolic compounds as reported in Table 4. As known, the composition of a phytoextract is closely related to the operative conditions which may influence the physicochemical properties of molecules to be extracted and, thus, their solubility. Furthermore, side reactions might occur based on the respective extraction methodology and solvent used, which can lead to structural rearrangements and alterations.

Despite the increased spectrophotometric results obtained by applying the ChCl:CA system to UAE for 40 min, a general reduction in the phenol concentration determined via HPLC-DAD was observed. The only exception was represented by caffeic acid, the slight increase in which could be attributed to an effective enhanced extraction via UAE or to a hydrolytic breaking of the ester bond in the chlorogenic acid as well as, conceivably, in its isomer. Therefore, it can be argued that the higher extraction power of the ultrasonic treatment for 40 min over the others is the result of a synergism between multiple compounds, other than the phenols in the mixture, capable of reacting in the free radical-scavenging mechanism.

**Figure 2:**
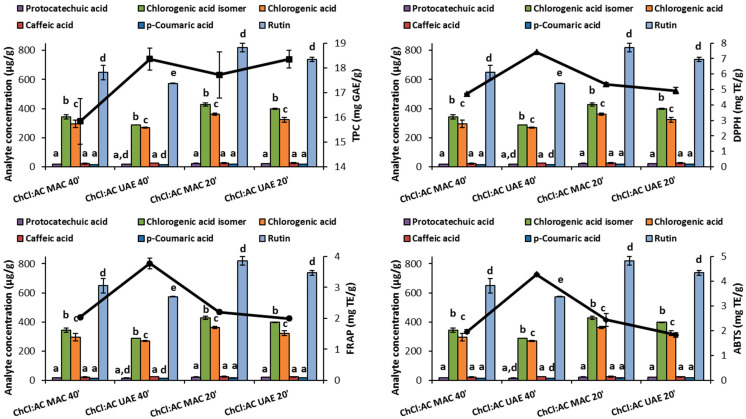
Trend of the phenol composition and antioxidant activity as the extraction conditions vary: the bars refer to the phenol identified via HPLC, while the lines refer to the specific spectrophotometric assay. Error bars indicate data variability for the reported measurements. Within each group corresponding to the specific extraction condition, different letters above the bars indicate a statistically significant difference at a *p*-value < 0.05 (*n* = 3).

What stands out from Table 4 is the higher concentration of all molecules detected when both MAC and UAE were carried out for 20 min, particularly evident for chlorogenic acid, its isomer and rutin. This result clearly emerges from Figure 2. For these compounds, statistically significant results (*p* < 0.05) were recorded when comparing the data obtained between 20 min and 40 min of extraction. This in turn highlights that longer extraction times could reasonably lead to possible degradations.

Regardless of the experiment type, the content of chlorogenic acid was higher than that reported by Palmieri et al. obtained with MAC and UAE and conventional hydro-alcoholic mixtures (27.82 ± 0.94 and 146.90 ± 0.83 µg/g DW, respectively) [10]. The content of caffeic acid, instead, was in line with that obtained using UAE reported by the same authors (27.88 ± 0.35 µg/g DW). Msaada et al. found a content of *p*-coumaric acid equal to 43.02 ± 5.26 (µg/g DW, in the Tunisia variety) and of rutin equal to 139.6 ± 14.26 (µg/g DW, in the Syria variety) in coriander seeds via an extraction with methanol for 30 min [36]. What stands out from our study is the extraordinarily high content of rutin: the great interest towards this molecule has been already evaluated with the preparation of rutin-enriched coriander extracts endowed with a radioprotective effect against hematopoietic injury, associated with reduced reactive oxygen species and enhanced enzymatic antioxidant activity [37].

When considering the relationship between the total peak content measured via HPLC-DAD vs. spectrophotometric assays, contradictory results between the data obtained using ChCl:CA for 40 min coupled to MAC and UAE resulted in an overall worsening in terms of correlations. In particular, it was observed that the experiment with MAC had a higher negative impact on such a correlation, in fact, its omission resulted in a significant improvement of the correlation between the HPLC outcomes and the four spectrophotometric assays (R^2^ HPLC-PC vs. TPC = 0.566; R^2^ _HPLC-PC vs. DPPH_ = 0.790; R^2^ _HPLC-PC vs. FRAP_ = 0.829; R^2^ _HPLC-PC vs. ABTS_ = 0.721).

In general, the strong correlation between HPLC-DAD and TPC suggests that this assay is reasonably valid for a rough prediction of the phenolic content in the coriander extracts. For this reason, the 20 min extraction time was selected for successive experiments.

### 3.3. Comparison of NADES Systems Coupled with MAC and UAE

The extractive performance provided by the ChCl:CA-based acidic system, in place of the hydro-alcoholic solvent, was compared with two NADES endowed with more neutral properties. Accordingly, with the aim of selecting the most appropriate NADES for the extraction of phenols from coriander seeds, urea (Ur) and glucose (Glu) were selected as hydrogen bond donors (HBDs) in combination with choline chloride as the HBA counterpart.

Tymczewska et al. have recently screened three NADES systems for their ability to extract phenolic compounds from spices, including coriander seeds [38]. Although the ChCl:Ur-based system was found to be a suboptimal solvent for the isolation of bioactives, the conventional hydro-methanolic solvent appeared to be more effective. As reported by the authors, this result paves the way for the potential of a solvent such as ChCl:Ur as a sustainable alternative in the extraction process, prior to tuning and optimizing its properties. Other studies have shown the excellent solvent properties of ChCl:Ur, i.e., for the extraction of valuable active compounds from the olive leaf [39], from *Achillea millefolium* L. [40] and from onion peel [41]. Analogously, the efficiency of sugar-based NADES has been reported in the literature. Choudhary et al. determined the extraction efficiency of different combinations of choline chloride with sugars and polyalcohols, including glucose, on *Ocimum sanctum*, *Terminalia bellerica* and *T. chebula* medicinal plants [42]. Among the screened NADES, the system with ChCl:Glu (2:1) was found to be the least effective in determining the total phenolic and flavonoid content as well as the ferric-reducing power. However, the results were generally higher than those provided by extracts prepared from methanol, in particular for *Terminalia bellerica* and *T. chebula*. In the study of Jeong et al., a UAE-NADES methodology applied for the simultaneous extraction and characterization of polar bioactive compounds and volatile monoterpenes from peppermint leaves, demonstrated the ChCl:Glu (5:2) system as the most promising solvent [43].

In this framework, the extraction efficiency of the phenolic compounds using the newly prepared NADES (ChCl:Ur and ChCl:Glu), not studied so far on coriander seeds, was compared in terms of the TPC and TAC value. Then, a quantitative HPLC-DAD analysis of the extracts was applied to monitor and compare the concentrations of the main phenolic compounds. This evaluation was performed under the optimized extraction conditions (20 min extraction time), in a one-step methodology with MAC or UAE. The results are reported in Table 5.

Notably, the improvement in the performance of spectrophotometric assays was evident for TPC and ABTS with the ChCl:Glu system as the extracting solvent and for the FRAP and ABTS results with ChCl:Ur. Intriguingly, in the presence of urea as the HBD, the increased levels of chlorogenic acid and its isomer could be responsible for the improved antioxidant capacity expressed as ferric reducing power (FRAP) and anti-radical activity (ABTS), while an analogous effect on the same assays was performed by protocatechuic, caffeic and *p*-coumaric acids when glucose was used as a NADES component (Table 6).

Noteworthy, what emerged from these results is that NADES were coupled in both cases with the UAE methodology. It is also plausible to deem that the high values obtained for rutin, chlorogenic acid and its isomer with the ChCl:Ur using UAE, compared to the other extraction conditions, could alter the trend of the results. In fact, their exclusion from the data correlation (considering the total content using HPLC vs. each spectrophotometric assay) resulted in a substantial improvement in the R^2^ factors (R^2^ _HPLC-PC vs. TPC_ = 0.993; R^2^ _HPLC-PC vs. DPPH_ = 1.0; R^2^ _HPLC-PC vs. FRAP_ = 0.914; R^2^ _HPLC-PC vs. ABTS_ = 0.879). As previously observed for the citric acid-based NADES, the use of ChCl:Ur and ChCl:Glu did not affect the spectrophotometric response.

**Figure 3:**
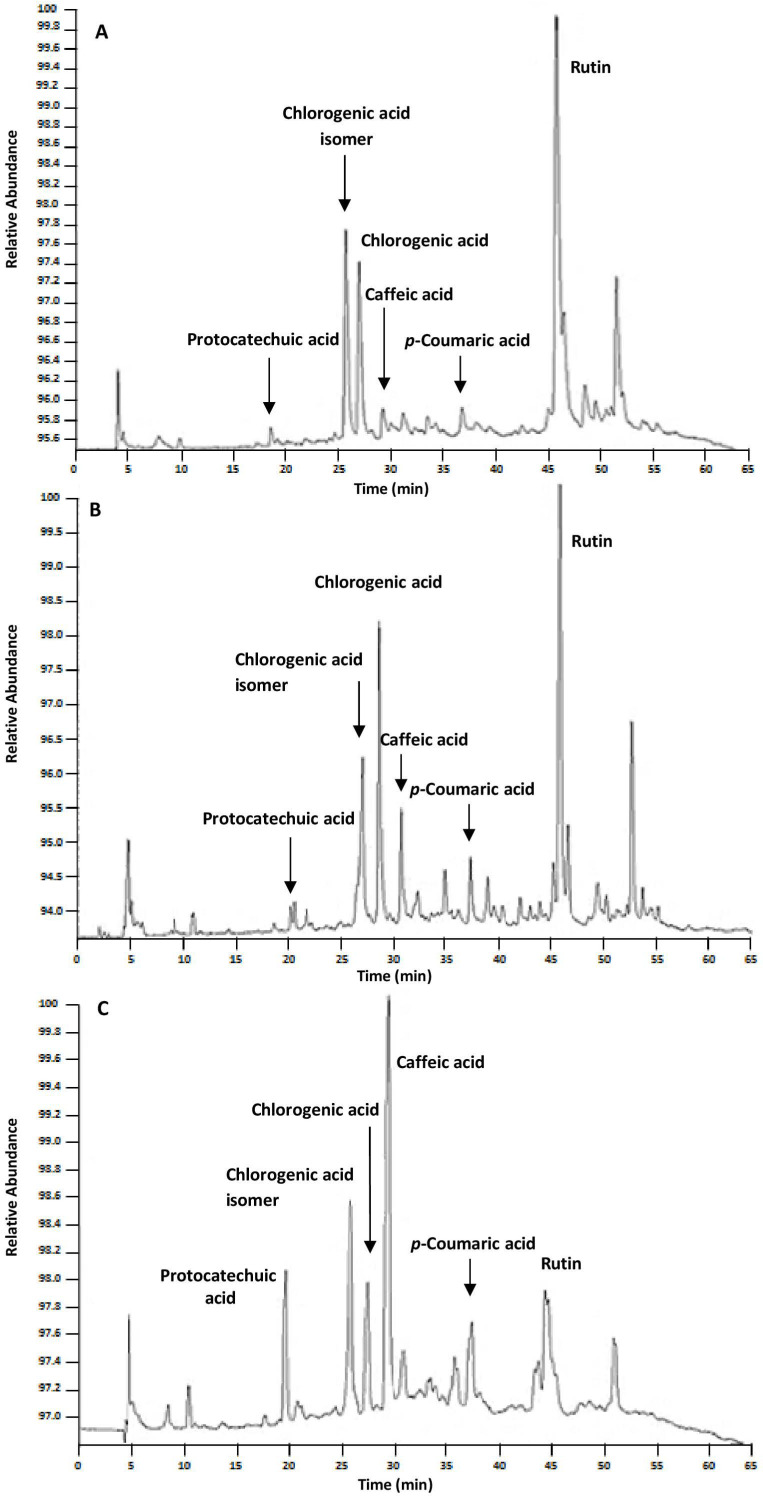
HPLC-DAD chromatograms of phenolic compounds extracted from coriander seeds using NADES solvents (**A**) ChCl:CA, (**B**) ChCl:Ur and (**C**) ChCl:Glu in a one-step UAE methodology, for 20 min.

It is also important to underline that the different properties (polarity, viscosity, density) of the NADES investigated in the present work did not alter the extractability in terms of the identity of the targeted compounds. In fact, the three NADES shared a common behaviour in the extraction selectivity of phenolic compounds as represented in the chromatographic profiles (Figure 3) where, as stated above, the different content of extracted bioactives made the difference. This highlights how the physicochemical properties of the tailored NADES modulated the extraction efficiency by triggering specific molecular interactions, the promotion or inhibition of which affect the solubility and stability of the solutes [44,45].

This finding could be fruitfully exploited by creating ad hoc NADES-based systems when the attention is focused on a leading compound whose obtainable amount needs to be enhanced. The use of CA or Ur-based NADES, respectively, coupled with MAC or UAE for 20 min, allowed us to obtain high amounts of chlorogenic acid, a compound that is gaining great attention for its outstanding pro-health properties including antioxidant, radical scavenging, anticancer, antiobesity and antidiabetic activity [2,23]. Through the same approaches, high levels of rutin (also known as vitamin P) were extracted, a flavonoid highly recognized for its pharmacological activities and variously used in food, cosmetic and chemical industries [46]. Instead, the system based on ChCl:Glu coupled with UAE for 20 min improved the levels of protocatechuic, caffeic and *p*-coumaric acids which exhibit many health benefits associated with their antioxidant and anti-inflammatory properties.

## 4. Conclusions

In the present work, for the first time, a systematic study dealing with the use of a NADES-aided system as an efficient solvent in the extraction of phenolic compounds from coriander seeds has been reported. The use of a choline chloride (ChCl)-based solvent, as a common hydrogen bond acceptor for NADES, in combination with different hydrogen bond donors [citric acid (CA), urea (Ur) and glucose (Glu)] was coupled with MAC or the non-conventional UAE technique in a one-step methodology. Optimizing the extraction process through a one-variable-at-a-time (OVAT) approach proved useful for gaining insights into the influence of independent process variables on the recovery of phenol compounds. Spectrophotometric assays (TPC, DPPH, FRAP and ABTS) as well as a chromatographic (HPLC-DAD) analysis allowed us to monitor and compare each step of the extraction process.

These preliminary findings demonstrated that the applied extraction conditions showed a higher extraction efficiency than the conventional hydro-methanolic mixture, thus corroborating the potential of the proposed strategy for the extraction of bioactives from coriander seeds. Moreover, it has been proven that a successful NADES-assisted extraction can be achieved in 20 min, making the entire extraction process sustainable and energy-saving. Noteworthy, the three systems based on ChCl:CA, ChCl:Ur and ChCl:Glu showed a similar extraction selectivity in terms of extractable molecules but a different extraction efficiency. In fact, ChCl:CA coupled with MAC for 20 min and ChCl:Ur coupled with UAE for 20 min allowed us to obtain high amounts of chlorogenic acid and its isomer, as well as rutin, while ChCl:Glu coupled with UAE for 20 min improved the levels of protocatechuic, caffeic and *p*-coumaric acids. This feature could be profitably exploited by modulating the NADES composition in order to tailor the extraction towards higher levels of a desirable bioactive.

The preliminary results obtained in our study encourage us to devote future studies to a larger number of NADES to test, as well as researching sustainable recovery approaches based on NADES recycling systems. This treatment would also be desirable to obtain NADES-free extracts in view of future biological evaluations.

According to our findings, coriander seeds were revealed to be a rich source of phenols, and the identification of the most influential extraction parameters, in a green and sustainable process perspective, is promising for potential industrial design and scale-up.

## Figures and Tables

**Table 1:** TPC and in vitro antioxidant activity measured with the DPPH, FRAP and ABTS assays of coriander extracts obtained via maceration (MAC) with methanol:water (70:30, *v*/*v*) as extraction solvent. Results, reported as mean value ± standard deviation (*n* = 3), are expressed on dry weight.

Coriander Extract	TPC (mg GAE/g)	DPPH (mg TE/g)	FRAP (mg TE/g)	ABTS (mg TE/g)
MAC 40 min	15.85 ± 0.69 ^a^	3.12 ± 0.10 ^a,b^	3.74 ± 0.04 ^a^	4.04 ± 0.12 ^a^
MAC 2 h	9.29 ± 0.04 ^b^	3.29 ± 0.25 ^a,b^	3.87 ± 0.13 ^a^	4.38 ± 0.02 ^a,b^
MAC 4 h	11.39 ± 0.32 ^c^	2.88 ± 0.29 ^a^	3.60 ± 0.03 ^a^	3.73 ± 0.17 ^b^
MAC 6 h	11.41 ± 0.48 ^c^	3.89 ± 0.23 ^b^	5.04 ± 0.01 ^b^	5.63 ± 0.08 ^c^

Different superscript letters in the same column indicate significant differences (*p*-value < 0.05).

**Table 2:** Quantitative HPLC-DAD results of the identified phenols in coriander extracts obtained via MAC with methanol:water (70:30, *v*/*v*) as extraction solvent. Results are reported as mean value ± standard deviation (*n* = 3) on dry weight of coriander.

Coriander Extract	Analyte Concentration (µg/g)					
	Protocatechuic Acid	Chlorogenic Acid Isomer	Chlorogenic Acid	Caffeic Acid	*p*-Coumaric Acid	Rutin
MAC 40 min	22.18 ± 0.18 ^a^	258.01 ± 18.72 ^a^	321.89 ± 20.94 ^a^	19.03 ± 0.32 ^a^	14.45 ± 1.21 ^a^	675.63 ± 9.62 ^a^
MAC 2 h	15.69 ± 0.54 ^b^	225.93 ± 8.76 ^a^	298.18 ± 9.89 ^a^	17.93 ± 1.59 ^a^	14.71 ± 1.15 ^a^	640.64 ± 3.50 ^a^
MAC 4 h	15.55 ± 0.12 ^b^	214.87 ± 0.24 ^a^	298.72 ± 4.65 ^a^	19.46 ± 1.18 ^a^	11.84 ± 0.55 ^a^	637.65 ± 25.22 ^a^
MAC 6 h	17.86 ± 1.47 ^b^	236.67 ± 5.89 ^a^	339.20 ± 9.42 ^a^	18.93 ± 3.47 ^a^	15.06 ± 0.89 ^a^	719.53 ± 26.11 ^a^

Different superscript letters in the same column indicate significant differences (*p*-value < 0.05).

**Table 3:** TPC and in vitro antioxidant activity measured with the DPPH, FRAP and ABTS assays of coriander extracts obtained us8ing MAC and ultrasound-assisted extraction (UAE) with the choline chloride–citric acid (ChCl:CA 1:1, *v*/*v*) NADES system. Results, reported as mean value ± standard deviation (*n* = 3), are expressed on dry weight.

Coriander Extract	TPC (mg GAE/g)	DPPH (mg TE/g)	FRAP (mg TE/g)	ABTS (mg TE/g)
ChCl:CA MAC 40 min	15.84 ± 0.92 ^a^	4.73 ± 0.04 ^a^	2.03 ± 0.05 ^a^	1.97 ±0.08 ^a^
ChCl:CA UAE 40 min	18.35 ± 0.44 ^a^	7.42 ± 0.00 ^b^	3.77 ± 0.17 ^b^	4.27 ± 0.01 ^b^
ChCl:CA MAC 20 min	17.72 ± 0.93 ^a^	5.35 ± 0.12 ^c^	2.21 ± 0.06 ^a^	2.43 ± 0.27 ^a^
ChCl:CA UAE 20 min	18.34 ± 0.36 ^a^	4.92 ± 0.20 ^a,c^	1.99 ± 0.03 ^a^	1.83 ± 0.09 ^a^

Different superscript letters in the same column indicate significant differences (*p*-value < 0.05).

**Table 4:** Quantitative HPLC-DAD results of the identified phenols in coriander extracts obtained via MAC and UAE with the ChCl:CA (1:1, *v*/*v*) NADES system. Results are reported as mean value ± standard deviation (*n* = 3) on dry weight of coriander.

Coriander Extract	Analyte Concentration (µg/g)					
	Protocatechuic Acid	Chlorogenic Acid Isomer	Chlorogenic Acid	Caffeic Acid	*p*-Coumaric Acid	Rutin
ChCl:AC MAC 40 min	18.82 ± 0.67 ^a,b^	343.76 ± 13.97 ^a^	294.189 ± 25.29 ^a^	21.25 ± 3.60 ^a^	16.07 ± 0.00 ^a^	648.18 ± 48.70 ^a,c^
ChCl:AC UAE 40 min	16.51 ± 0.18 ^a^	288.29 ± 0.71 ^b^	269.33 ± 4.45 ^a^	24.33 ± 0.28 ^a^	15.05 ± 0.25 ^a^	573.64 ± 2.54 ^a^
ChCl:AC MAC 20 min	23.56 ± 2.55 ^b^	428.50 ± 10.89 ^c^	361.33 ± 7.27 ^b^	25.92 ± 2.58 ^a^	17.94 ± 1.45 ^a^	820.31 ± 28.59 ^b,c^
ChCl:AC UAE 20 min	21.72 ± 1.01 ^a,b^	398.18 ± 2.39 ^c^	323.11 ± 15.28 ^a,b^	24.10 ± 3.45 ^a^	17.72 ± 0.42 ^a^	736.85 ± 15.04 ^c^

Different superscript letters in the same column indicate significant differences (*p*-value < 0.05).

**Table 5:** TPC and in vitro antioxidant activity measured with the DPPH, FRAP and ABTS assays of coriander extracts obtained by MAC and UAE with the choline chloride-urea (ChCl:Ur 1:1, *v*/*v*) and choline chloride-glucose (ChCl:Glu 1:1, *v*/*v*) systems. Results, reported as mean value ± standard deviation (*n* = 3), are expressed on dry weight.

Coriander Extract	TPC (mg GAE/g)	DPPH (mg TE/g)	FRAP (mg TE/g)	ABTS (mg TE/g)
ChCl:Ur MAC 20 min	17.47 ± 1.16 ^a^	3.77 ± 0.04 ^a^	3.26 ± 0.11 ^a^	7.75 ± 0.21 ^a^
ChCl:Ur UAE 20 min	21.43 ± 0.16 ^a^	4.66 ± 0.12 ^b^	4.79 ± 0.05 ^b^	11.46 ± 0.13 ^b^
ChCl:Glu MAC 20 min	29.11 ± 2.20 ^b^	3.68 ± 0.19 ^a^	3.79 ± 0.06 ^c^	8.58 ± 0.15 ^c^
ChCl:Glu UAE 20 min	34.17 ± 0.55 ^b^	3.93 ± 0.20 ^a^	4.31 ± 0.04 ^d^	9.58 ± 0.14 ^d^

Different superscript letters in the same column indicate significant differences (*p*-value < 0.05).

**Table 6:** Quantitative HPLC-DAD results of the identified phenols in coriander extracts obtained via MAC and UAE with the ChCl:Ur (1:1, *v*/*v*) and ChCl:Glu (1:1, *v*/*v*) systems. Results are reported as mean value ± standard deviation (*n* = 3) on dry weight of coriander.

Coriander Extract	Analyte Concentration (µg/g)					
	Protocatechuic Acid	Chlorogenic Acid Isomer	Chlorogenic Acid	Caffeic Acid	*p*-Coumaric Acid	Rutin
ChCl:Ur MAC 20 min	39.95 ± 0.26 ^a,b^	370.09 ± 2.59 ^a^	335.18 ± 14.63 ^a^	65.51 ± 5.14 ^a^	23.84 ± 0.38 ^a^	592.94 ± 9.00 ^a^
ChCl:Ur UAE 20 min	30.68 ± 0.36 ^a^	537.42 ± 1.27 ^b^	453.90 ± 4.77 ^b^	93.70 ± 0.03 ^b^	30.94 ± 0.05 ^b^	766.25 ± 4.85 ^b^
ChCl:Glu MAC 20 min	50.12 ± 1.41 ^b^	265.42 ± 3.04 ^c^	231.06 ± 1.39 ^c^	155.57 ± 7.02 ^c^	38.27 ± 0.40 ^c^	380.73 ± 2.81 ^c^
ChCl:Glu UAE 20 min	131.13 ± 6.16 ^c^	184.81 ± 8.60 ^d^	152.63 ± 10.06 ^d^	269.03 ± 4.15 ^d^	57.36 ± 0.06 ^d^	235.09 ± 2.19 ^d^

Different superscript letters in the same column indicate significant differences (*p*-value < 0.05).

## Data Availability

The data presented in this study are available in this manuscript.

## Supplementary Materials

The following supporting information can be downloaded at: https://www.mdpi.com/article/10.3390/antiox12122048/s1, Table S1: Calibration data: regression equation, linearity range, coefficient of determination value (R2), LOD and LOQ values; Table S2: Method validation: evaluation of precision (RSD %) and accuracy (recovery %) in the short- and long-term period (intra-day and inter-day precision and accuracy values); Figure S1: IR spectrum of choline chloride; Figure S2: IR spectrum of glucose; Figure S3: IR spectrum of ChCl:Glu; Figure S4: IR spectrum of urea; Figure S5: IR spectrum of ChCl:Ur; Figure S6: IR spectrum of citric acid; Figure S7: IR spectrum of ChCl:CA.
