# Supplementary material for: NADES-Assisted Extraction of Polyphenols from Coriander Seeds: A Systematic Optimization Study
Source: Antioxidants (Basel). 2023 Nov 27;12(12):2048. doi: 10.3390/antiox12122048 (PMC10741060; doi:10.3390/antiox12122048)
Supplement: Supplementary file 1 [file antioxidants-12-02048-s001.zip › antioxidants-2669616-supplementary.pdf]

Supplementary Material

NADES-Assisted Extraction of Polyphenols from Coriander Seeds: A Systematic Optimization Study

Federica Ianni [1,*], Samir Scandar [2], Luciano Mangiapelo [1], Francesca Blasi [1], Maria Carla Marcotullio [2,*] and Lina Cossignani [1]

[1] Department of Pharmaceutical Sciences, Section of Food, Biochemical, Physiological and Nutrition Sciences, University of Perugia, 06126 Perugia, Italy; luciano.mangiapelo@studenti.unipg.it (L.M.); francesca.blasi@unipg.it (F.B.); lina.cossignani@unipg.it (L.C.)

[2] Department of Pharmaceutical Sciences, Section of Morphological, Biomolecular, Nutraceutical and Health Sciences (SIMBIONUS), University of Perugia, 06122 Perugia, Italy; samir.scandar@studenti.unipg.it

* Correspondence: federica.ianni@unipg.it (F.I.); mariacarla.marcotullio@unipg.it (M.C.M.); Tel.: +39-075-585-7955 (F.I.); +39-075-585-7215 (M.C.M.)

Table S1. Calibration data: regression equation, linearity range, coefficient of determination value (R^2), LOD and LOQ values.

Cpd	Regression Equation	Linearity range (μg/mL)	R^2	LOD* (ng/mL)	LOQ* (ng/mL)
**Protocathecuic acid**	y = 34382.41(±984.34)x + 1043.08(±127.25)	0.25-2.5	0.9992	0.012	0.037
**Chlorogenic acid**	y = 27274.77(±821.48)x + 4021.01(±206.79)	0.25-5.0	0.9986	0.025	0.075
**Caffeic acid**	y = 60206.50(±1496.30)x + 7648.416(±376.67)	0.25-5.0	0.9991	0.021	0.063
***p*-Coumaric acid**	y = 55066.02(±4144.86)x + 13737.04(±1043.40)	0.25-5.0	0.9992	0.063	0.189
**Rutin**	y = 33579.30(±866.19)x - 26486.17(±1213.44)	2.5-25	0.9990	0.119	0.361

*LOD and LOQ values were calculated as the standard deviation of the response (σy) on the slope of the calibration curve (b), according to the equations: C_{LOD}= 3.3(σy/b) and C_{LOQ} = 10(σy/b)

Table S2. Method validation: evaluation of precision (RSD %) and accuracy (Recovery %) in the short- and log-term period (intra-day and inter-day precision and accuracy values).

Nominal Conc. (μg/mL)	Intra-day mean concentration (μg/mL)			Intra-day mean Precision (RSD%)			Intra-day Accuracy (Recovery)		
	**Chlorogenic acid**	**Caffeic acid**	***p*-Coumaric acid**	**Chlorogenic acid**	**Caffeic acid**	***p*-Coumaric acid**	**Chlorogenic acid**	**Caffeic acid**	***p*-Coumaric acid**
3.3	33.63	33.95	33.45	0.71	2.30	1.16	101.90	102.89	101.35
	33.84	33.45	33.32	1.67	2.66	1.91	102.54	101.37	100.97
	32.36	33.41	33.75	3.44	3.37	1.71	98.07	101.24	102.27
33	3.32	3.27	3.32	1.83	2.36	1.08	100.68	99.11	100.67
	3.25	3.33	3.34	1.69	1.85	3.23	98.42	100.86	101.10
	3.24	3.41	3.37	1.27	2.88	0.17	98.11	103.31	102.08

Nominal Conc. (μg/mL)	Inter-day mean concentration (μg/mL)			Inter-day Precision (RSD%)			Inter-day Accuracy (Recovery%)		
	**Chlorogenic acid**	**Caffeic acid**	***p*-Coumaric acid**	**Chlorogenic acid**	**Caffeic acid**	***p*-Coumaric acid**	**Chlorogenic acid**	**Caffeic acid**	***p*-Coumaric acid**
3.3	33.28	33.60	33.50	2.82	2.55	1.52	100.84	101.83	101.53
33	3.27	3.34	3.34	1.86	2.76	1.81	99.07	101.09	101.28

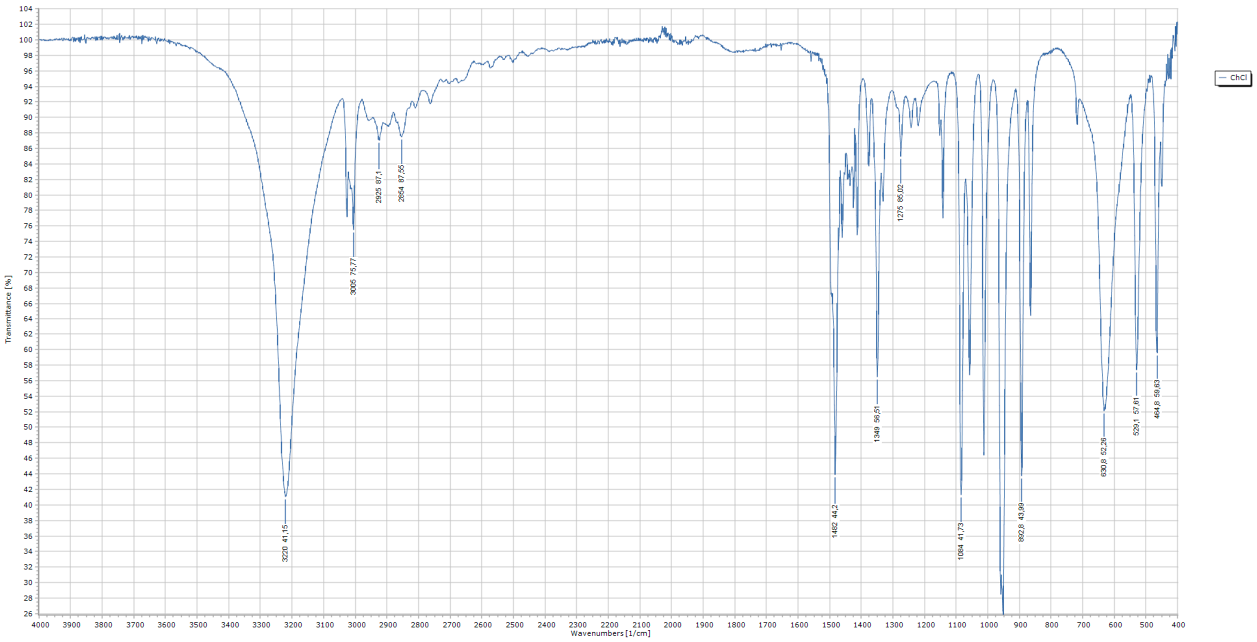

Figure S1. IR spectrum of Choline chloride

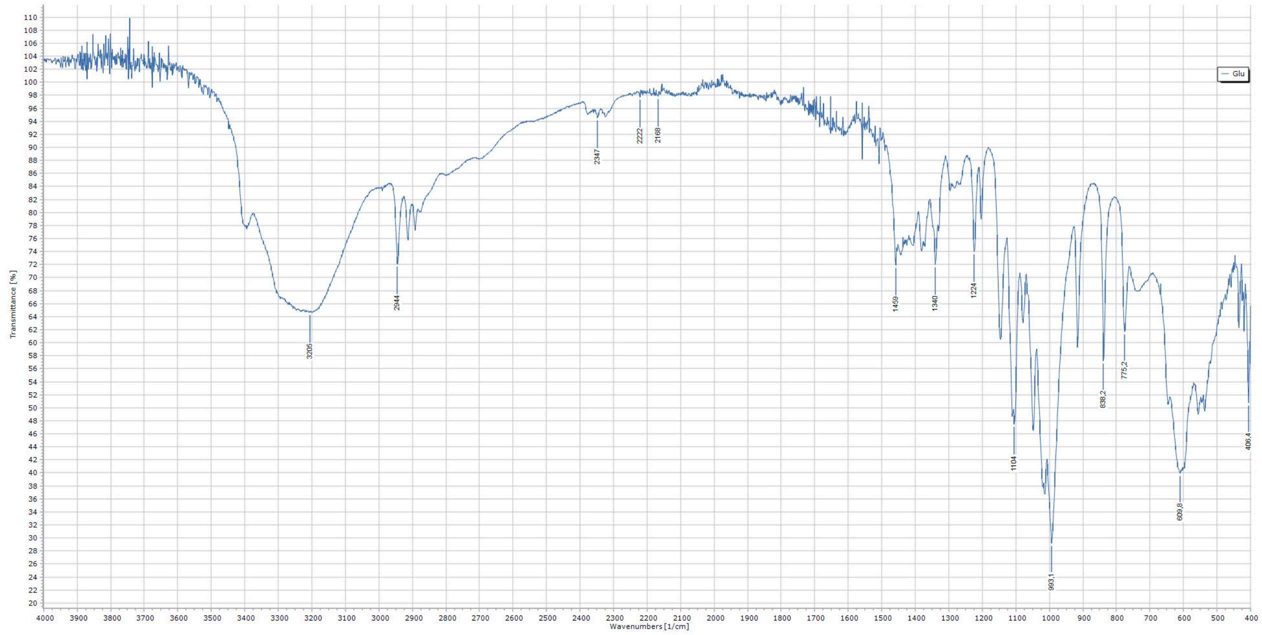

Figure S2. IR spectrum of Glucose

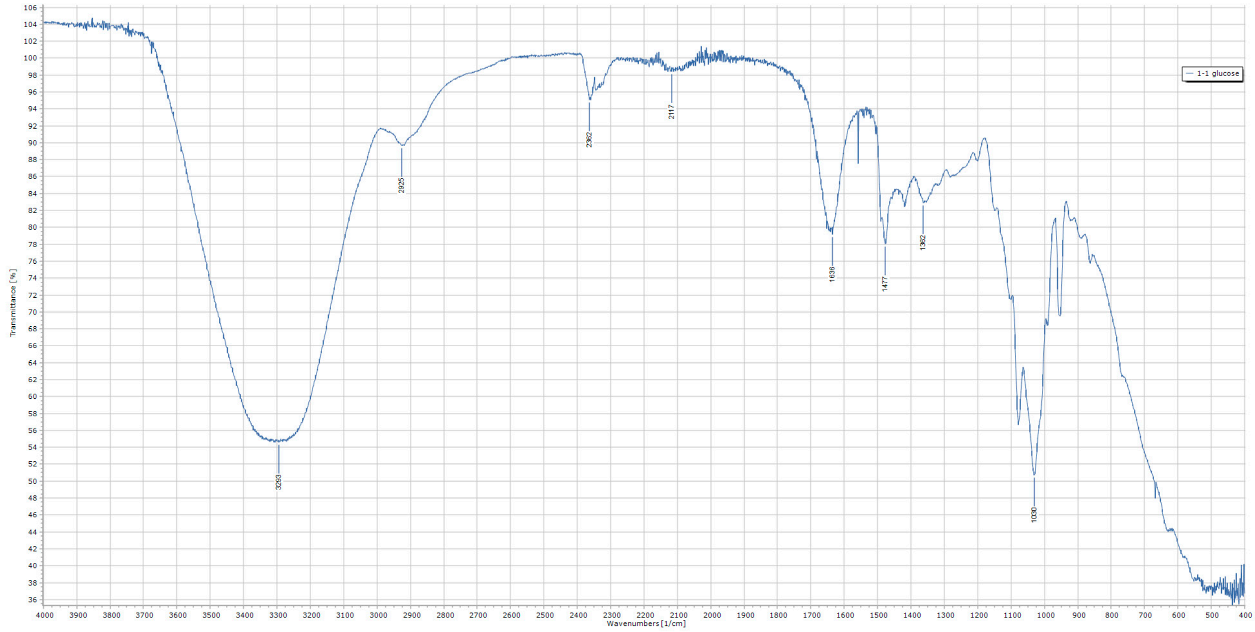

Figure S3. IR spectrum of ChCl:Glu

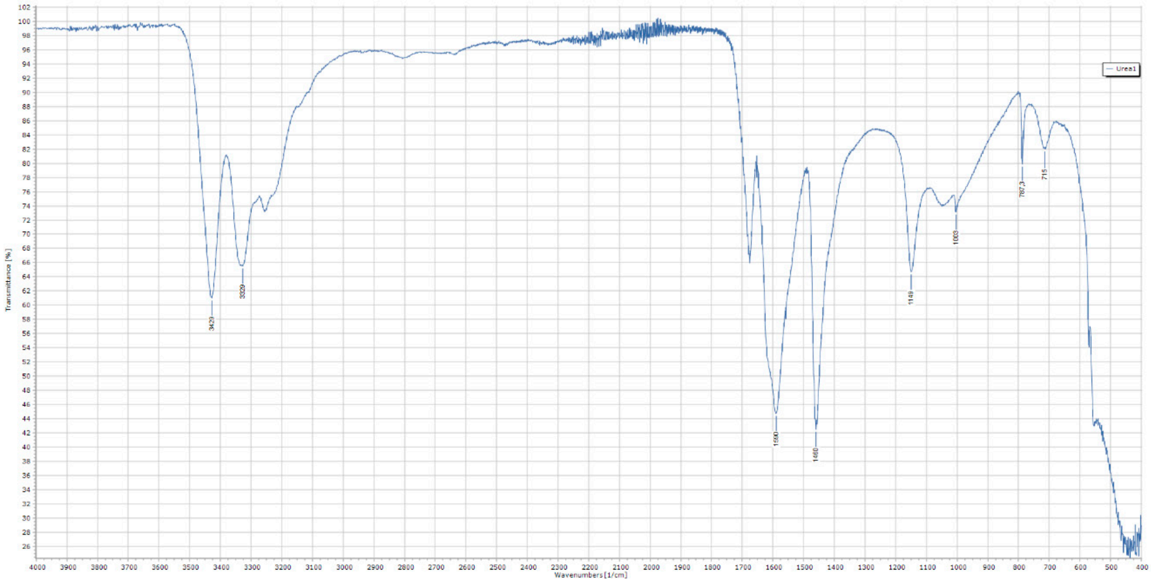

Figure S4. IR spectrum of Urea

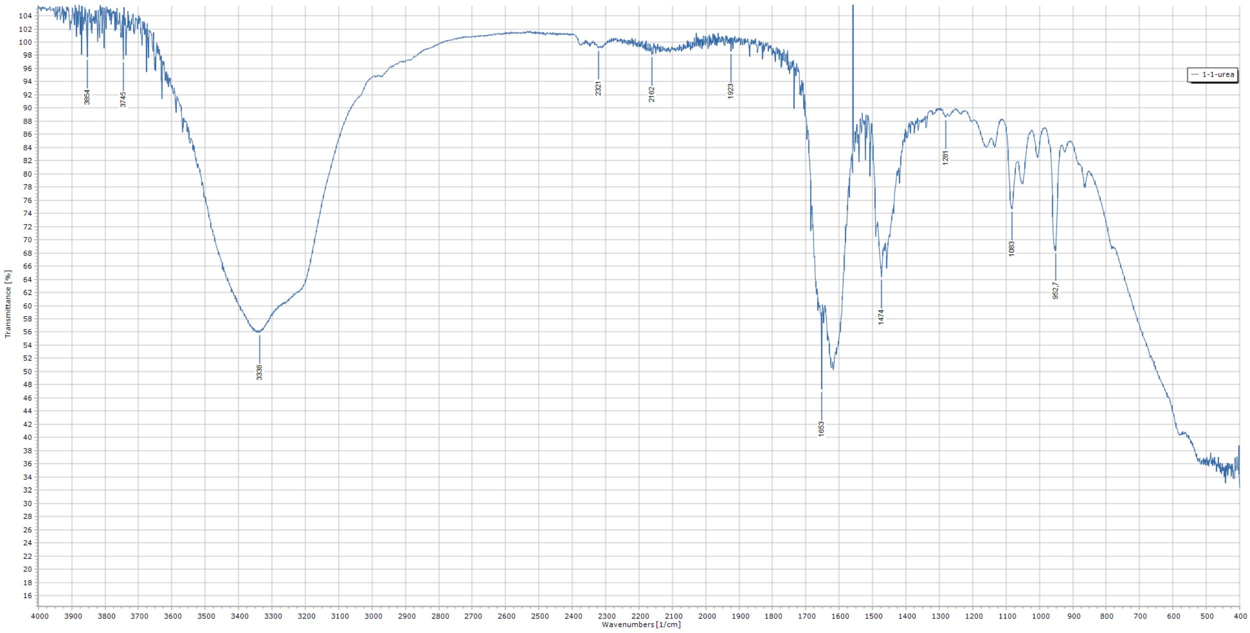

Figure S5. IR spectrum of ChCl:Ur

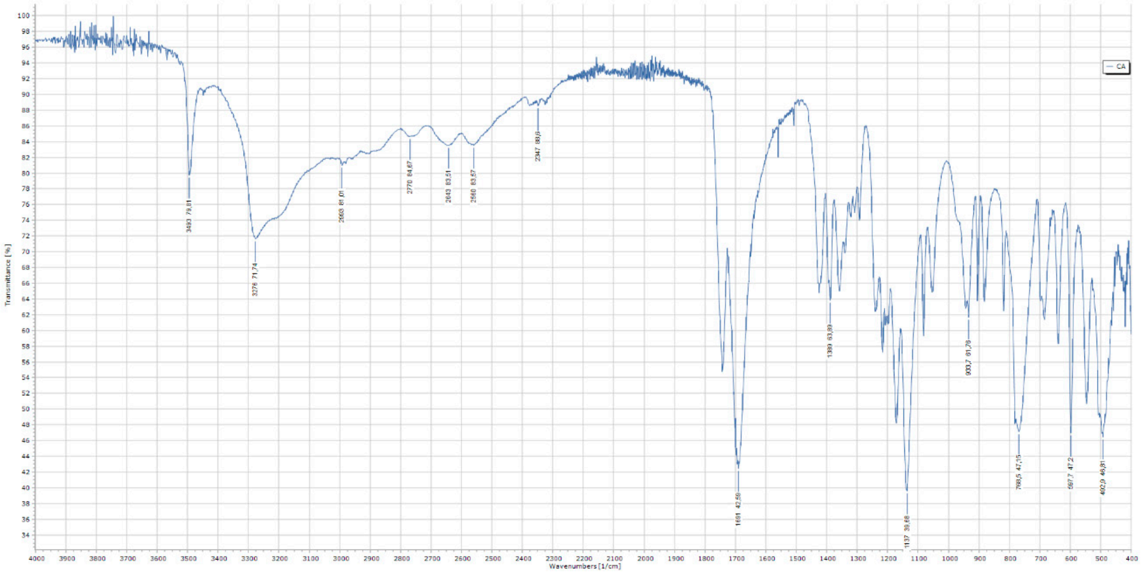

Figure S6. IR spectrum of Citric acid

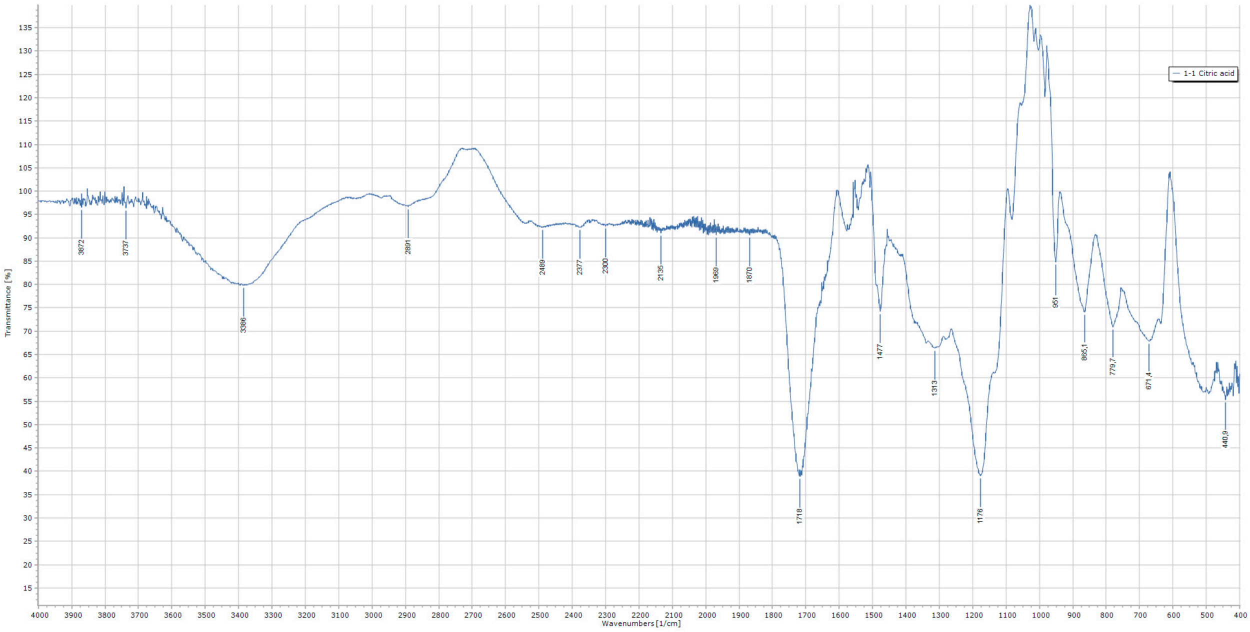

Figure S7. IR spectrum of ChCl:CA
